# Of Drugs and Trypanosomatids: New Tools and Knowledge to Reduce Bottlenecks in Drug Discovery

**DOI:** 10.3390/genes11070722

**Published:** 2020-06-29

**Authors:** Arijit Bhattacharya, Audrey Corbeil, Rubens L. do Monte-Neto, Christopher Fernandez-Prada

**Affiliations:** 1 Department of Microbiology, Adamas University, Kolkata, West Bengal 700 126, India; arijbhatta@gmail.com; 2 Department of Pathology and Microbiology, Faculty of Veterinary Medicine, Université de Montréal, Saint-Hyacinthe, QC J2S 2M2, Canada; audrey.corbeil@umontreal.ca; 3 Instituto René Rachou, Fundação Oswaldo Cruz, Belo Horizonte MG 30190-009, Brazil; rubens.monte@fiocruz.br

**Keywords:** trypanosomatids, neglected tropical diseases, *Leishmania*, *Trypanosoma cruzi*, *Trypanosoma brucei*, drug discovery, in vitro models, in vivo models, genomics, drug resistance

## Abstract

Leishmaniasis (*Leishmania* species), sleeping sickness (*Trypanosoma brucei*), and Chagas disease (*Trypanosoma cruzi*) are devastating and globally spread diseases caused by trypanosomatid parasites. At present, drugs for treating trypanosomatid diseases are far from ideal due to host toxicity, elevated cost, limited access, and increasing rates of drug resistance. Technological advances in parasitology, chemistry, and genomics have unlocked new possibilities for novel drug concepts and compound screening technologies that were previously inaccessible. In this perspective, we discuss current models used in drug-discovery cascades targeting trypanosomatids (from in vitro to in vivo approaches), their use and limitations in a biological context, as well as different examples of recently discovered lead compounds.

## 1. Introduction: Status and Impact of Trypanosomatid-Borne Infections

In 1970, the Rockefeller Foundation coined the term “Neglected Tropical Diseases” (NTDs), which still applies to three major, chronic, debilitating, and poverty-promoting diseases caused by trypanosomatid parasites: human African trypanosomiasis (HAT or sleeping sickness), caused by *Trypanosoma brucei* and transmitted by tsetse flies; Chagas disease (South American trypanosomiasis) caused by *T. cruzi* and transmitted by blood-sucking triatomine bugs; and leishmaniasis, caused by various species of the genus *Leishmania* and transmitted by sand flies. At present, the therapeutic arsenal to combat these infections is ineffective and highly toxic. Progressively over the last two decades, this situation has been aggravated by the emergence and spread of drug-resistant strains [1]. 

**Figure 1:**
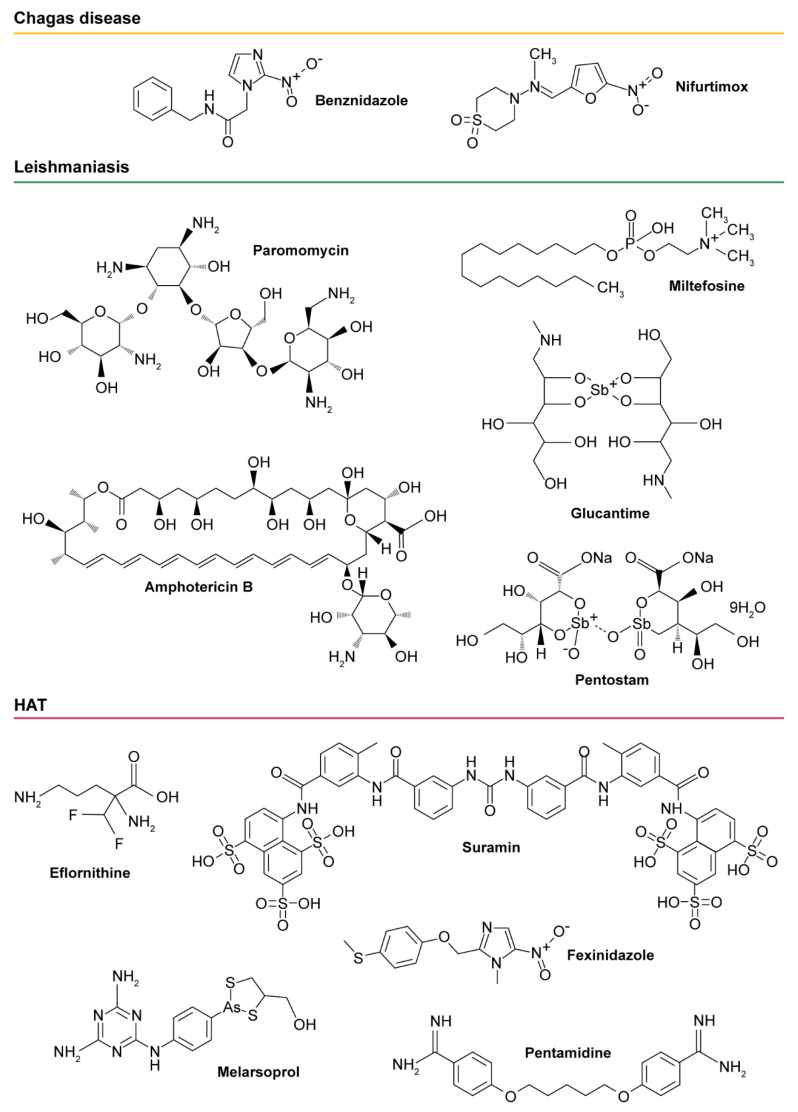
Drugs in clinical use against Chagas disease, leishmaniasis, and human African trypanosomiasis (HAT).

Although the WHO has targeted the elimination of HAT as a public health problem by 2020 (and interruption of transmission for 2030), Chagas disease and leishmaniasis are global threats in continuous expansion [2,3,4,5,6]. Chagas disease affects an estimated 8–10 million people worldwide, approximately 30% of which will develop chronic Chagas cardiac disease, leading to 14,000 deaths per year [1,6]. The cost of Chagas disease was estimated in 2013 at more than US$ 7 B/year, including lost productivity [7]. However, and despite these alarming numbers, only two toxic, old-fashioned compounds, benznidazole and nifurtimox (Figure 1), are approved for the treatment of Chagas disease [6,8]. While benznidazole is only FDA-approved for pediatric and acute cases of *T. cruzi* infection, nifurtimox is still only available under compassionate-use directives from the CDC [9,10]. Moreover, the efficacy of benznidazole treatment in chronic Chagas patients is controversial [10,11]. In addition to the unacceptable side effects of these drugs, drug resistance has emerged as a major concern in terms of treatment failure [1,12,13]. 

Leishmaniasis is estimated to be the ninth largest disease burden among individual infectious diseases, and the most dangerous of the NTDs. Leishmaniasis currently infects around 12 million people worldwide, and it is spreading with ca. 0.7–1 million new cases per year [14]. Dramatically, its visceral form (also referred as VL) has a 95% fatality rate among the poorest people in the world. The control of leishmaniasis relies on old-fashioned, highly toxic chemotherapy using a very limited number of registered molecules (Figure 1). In addition to toxicity, significant drawbacks such as complex route of administration, length of treatment, emergence of drug resistance, and costs limit their use in endemic areas [1,14]. Furthermore, NTDs are becoming emergent diseases in non-tropical countries, triggering vast socioeconomic consequences. The absence of investment to combat NTDs is likely due to their traditional cause of misfortune to poor, rural, and otherwise marginalized populations. However, their impact has shifted because of resistant strains and globalization. Without effective new drugs, the incidence of Chagas disease and leishmaniasis is expected to spread owing to climate change, global urbanization, immunosuppressive disease, etc. [15,16].

Traditionally, pharmaceutical companies have shown a very limited interest in improving current therapeutics against trypanosomatid parasites because of the expected low return on investment when targeting communities with little to no purchasing power [17,18]. In order to alleviate the costs and accelerate the marketing process [19,20,21] (e.g., to avoid obstacles during clinical trials, such as drug toxicity or unfavorable pharmacokinetics) [22], many initiatives are trying to find new indications for already-existing drugs, also known as drug repurposing (or drug repositioning) [1]. On the other hand, other initiatives—especially those stemming from academia—are targeted for identifying new points of intervention and to conceive novel drugs. In both cases, interdisciplinary research between experts in parasitology and chemistry is required, such that the former focus primarily on established drugs to treat infection due to limited access to novel molecules. Markedly, the critical situation with NTDs calls for the urgent development of high-throughput approaches for assessing drug efficacy and resistance, as well as novel therapeutics to avoid the emergence and spread of drug-resistant strains. Through this review, we aim to bring together these two major fields of knowledge and shed some light on the different models that are currently available, in order to build a drug-discovery pipeline targeting trypanosomatids (from in vitro to in vivo approaches), their use and limitations, as well as recent endeavors for discovering lead compounds.

## 2. Trypanosomatids′ Life Cycle in the Context of In Vitro Screening Assays

**Figure 2:**
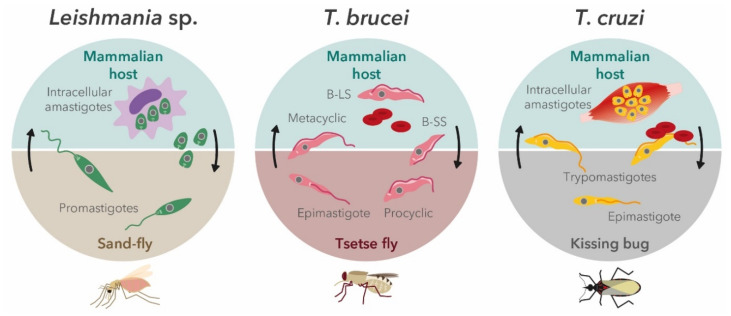
Life cycles of pathogenic trypanosomatid parasites. The clinically relevant life-cycle stages that are targets for drug intervention are intracellular amastigotes in *Leishmania* sp.; bloodstream forms (bloodstream long slender form (B-LS) and bloodstream short stumpy form (B-SS)) in *Trypanosoma brucei*; and infective trypomastigotes and intracellular amastigotes in *Trypanosoma cruzi*.

Pathogenic trypanosomatids have complex, digenetic lifecycles, which require the presence of both invertebrate and vertebrate hosts (summarized in Figure 2). In this way, various developmental stages throughout trypanosomatids’ lifecycle are required to guarantee their survival and spread. 

These diverse stages encompass many metabolic, biochemical, and cell biological adaptations, including a significant variation of cell morphology [23,24,25]. Because of these changes, it is hard, and sometimes impossible, to establish a correlation between compounds selected in assays targeting different forms of the same parasite (e.g., extracellular vs. intracellular). In the current lack of methodology standardization, this section will discuss the mains aspects to be considered to choose the most adapted in vitro screening assay to start a drug discovery cascade.

### 2.1. Leishmania Parasites

*Leishmania* parasites cycle between the motile promastigote form in the gut of the sand-fly vector and the intracellular amastigote stage within the macrophages and other types of mononuclear phagocytic cells of the mammalian host. In this way, when invading macrophages, *Leishmania* promastigotes block the phagosome maturation process and create an environment that is propitious to amastigote differentiation. Subsequent divisions and later infection of other mononuclear phagocytic cells, as well as different tissues, leads to the setup and progression of the clinical manifestations related to these diseases [26]. Traditionally, compounds have been evaluated by means of cell-free assays using axenic promastigotes and amastigotes, which allow high-throughput screening and high reproducibility, while relying on a limited number or parasites per evaluation. However, these two parasite forms present several important caveats that can lead to the selection of false candidates. On the one hand, promastigotes are not the mammalian form, and they show significant differences in their metabolic profile when compared to intracellular amastigotes. Moreover, their growth and sensitivity are influenced by different parameters, such as cell culture density, medium composition, and compound mode of action (MoA), among others, so care must be taken in interpreting the data [27]. While closer to the mammalian form, axenic amastigotes retain some promastigote traits, leading to a lack of correlation between axenic forms screenings and intracellular amastigote assays, which increases the false-positive rate of hit discovery when using this artificial form [28]. Consequently, models using the intracellular amastigote infecting mammalian host cells remain the gold standard in the determination of drug sensitivity. These models have great advantages such as the direct evaluation of drug penetration in the host cell, as well as drug activity in the phagolysosome milieu, among others [29,30]. Moreover, intracellular amastigotes are generally more sensitive than promastigotes against most of the drugs currently used in clinic, such as antimony or miltefosine [31,32], which could be a consequence of genes differentially regulated in the two developmental stages of the parasite [31,33,34]. The activity of candidate compounds against intracellular amastigotes is determined by microscopic automatic/manual counting of infected macrophages and the number of parasites per macrophage (parasitic index) or by spectrophotometric (e.g., optical density or staining) and fluorometric methods. These latter include the automated detection and quantification of genetically engineered amastigotes that express fluorescent and bioluminescent reporters, which enables faster read-outs and higher throughput [35]. Nonetheless, determination of the cidal and static effects of candidate compounds against intracellular forms can be very challenging, in part because of the slow replication rate of amastigotes when compared to promastigotes [36,37,38]. Moreover, this determination could be biased by many confounding factors that can reduce lab-to-lab reproducibility and lead to false hit discoveries. These factors could include macrophage infection rate, incomplete amastigogenesis, impact of distinct culture media, as well as the intrinsic pathogenicity of the strain selected for the assay [39,40,41]. 

Despite these potential limitations, in vitro amastigote assays (infecting THP-1 and primary mouse macrophages (PMM cells)) have led to the discovery and optimization of a novel series of amino-pyrazole ureas with potent antileishmanial activity [42]. Likewise, more recently, Van den Kerkhof et al. (2018) evaluated three antileishmanial leads series (nitroimidazoles, oxaboroles and aminopyrazoles) using intracellular *L. donovani* and *L. infantum* amastigotes infecting PMM, and showed a good in vitro to in vivo correspondence, with high efficacy and negligible side effects in vivo [43]. Tunes et al. (2020) found that gold(I)-derived complexes were very active against *L. infantum* and *L. braziliensis* intracellular amastigotes infecting THP-1 cells, including antimony-resistant strains (SbR), and they were potent inhibitors of trypanothione reductase. Moreover, two of these complexes presented very favorable pharmacokinetic and safety profiles in vivo after oral administration [44]. In the search of more robust, scalable, and reproducible models, Melby′s team developed an ex vivo splenic explant assay that allows the identification of new compounds active against *Leishmania* within the pathophysiologic environment [45,46]. In this way, they recovered the spleens of hamsters infected with a luciferase-transfected *L. donovani* strain, and used amastigote-harboring splenocytes to evaluate the antileishmanial activity of more than 4000 molecules. This medium-throughput screen revealed 84 small molecules with good antileishmanial activity and an acceptable toxicity evaluation [45]. Similarly, in a drug repurposing initiative, Fernandez-Prada et al. (2013) used BALB/c-derived splenic explants infected with *L. infantum* amastigotes expressing the infrared fluorescent protein IFP1.4 to evaluate the antileishmanial effect of anticancer-drug camptothecin and several analogues [37]. Markedly, and despite their many advantages, engineered parasites are not flawless, and different mitigation strategies should be taken into account in order to avoid any compensatory change in parasite metabolism or virulence (e.g., prioritize the use of integrative strategies to generate the strain) [35]. A final important remark is that, as has been recently demonstrated, there could be different compound efficiencies linked to the drug susceptibility background of the *Leishmania* strains used in the screening process (especially in the case of antimony susceptibility), which shows the potential value of including clinical isolates (and resistant strains) in the drug discovery cascade [47]. 

### 2.2. Trypanosoma brucei

Contrary to *Leishmania*, the *T. brucei* life cycle does not require the intracellular environment for any of its developmental forms. *T. brucei* is transmitted between mammalian hosts by *Glossina* spp. (tsetse fly), in which the bloodstream short stumpy form (B-SS) differentiates into the replicative procyclic form (PFs). PFs migrate to the proventriculus were they subsequently differentiate into epimastigotes and into cycle-arrested metacyclics (infective form) in the salivary glands of the tsetse fly. Parasites colonize the mammalian host during the blood meal of the fly and differentiate into bloodstream long slender form (B-LS), which eventually evolves to the B-SS form by a quorum-sensing mechanism [48,49]. Consequently, drug-screening assays targeting *T. brucei* rely on the bloodstream form of the parasite. Different approaches for whole-cell, high-throughput screening have recently been successfully developed. Mackey et al. (2006) screened 2160 FDA-approved drugs, bioactive compounds, and natural products to identify hits that were cytotoxic to *T. brucei* at a concentration of 1 μM or less. This approach led to the identification of 35 new hits from seven different drug categories, which included two approved trypanocidal drugs, suramin and pentamidine [50]. Similar to *Leishmania*, bioluminescent-engineered *T. brucei* have recently been developed and implemented in whole-cell high-throughput screens. Sykes et al. (2009) developed a luciferase-based viability assay for ATP detection in a 384-well format, making high-throughput whole-cell screening in *T. brucei* very reproducible, sensitive, and cost effective [51]. Later, Sykes et al. (2012) described the application of an Alamar Blue (resazurin)-based, 384-well high-throughput screening (HTS) assay to screen a library of 87,296 compounds, leading to 6 hits from 5 new chemical classes displaying great activity against *T.b. rhodesiense* [52]. As an alternative to luciferase and Alamar Blue, Faria et al. (2015) developed a whole-cell assay in 384-well plates based on the quantitative detection of double-stranded DNA bound to cyanine dye SYBR Green. The assay was a validated screening of a kinase-focused library composed of 4000 compounds, leading to the discovery of novel scaffolds with potent antitrypanosomal activity [53]. In the recent years, thanks to different screening initiatives, several new leads such as diamidine derivatives, fexinidazole, oxaborole SCYX-7158, quinolone amide GHQ168, and acoziborole are now in various stages of the development pipeline for treating HAT [54,55,56].

### 2.3. Trypanosoma cruzi

Infective trypomastigotes and intracellular replicative amastigotes are the clinically relevant life-cycle stages of *T. cruzi* that are targets for drug intervention [57]. Briefly, non-dividing *T. cruzi* metacyclic trypomastigotes are transmitted to humans in the feces of infected triatomine bugs at the bite site of these hematophagous insects. Trypomastigotes invade various cell types and transform into intracellular amastigotes, which multiply by binary fission until the host cell is overwhelmed, and then transform into bloodstream trypomastigotes and spread to distant sites through the lymphatics and bloodstream. Once back in the insect vector, trypomastigotes transform into epimastigotes and then differentiate into infective metacyclic trypomastigotes [58]. Despite many efforts, only two compounds, benznidazole (since 1972) and nifurtimox (since 1967), are currently used for the treatment of certain forms of Chagas disease [59]. Markedly, drug discovery in *T. cruzi* is handicapped by the small number of well-established targets (e.g., the sterol biosynthetic pathway, cruzipain, cytochrome b, trypanothione reductase, cyclophilin, or carbonic anhydrases [57]), which explains the wide use of phenotypic approaches that have become the main pillar of Chagas R&D [60]. Drug screening against *T. cruzi* can be performed in cell-free axenic amastigotes and epimastigotes, as well as in intracellular amastigotes, with similar advantages and caveats to those previously discussed for *Leishmania*. In terms of tools for measuring the trypanocidal effect of the compounds, screening systems have evolved from manual microscopic counting of parasite growth; the use of colorimetric substrates (e.g., chlorophenol-red-β-D-galactopyranoside); bioluminescent (e.g., parasites expressing the firefly luciferase) and fluorescent reporters (e.g., tdTomato-expressing lines); and high-content imaging approaches that do not require the incorporation of any reporter molecule [35,61,62]. Engel et al. (2010) developed a cell-based HTS assay that can be used with untransfected *T. cruzi* isolates and host cells that can simultaneously measure efficacy against the parasite and host cell toxicity. This approach was used to screen a library of 909 bioactive compounds, leading to the identification of 55 hits [63]. Using NIH-3T3 fibroblasts infected with a recombinant *T. cruzi* strain expressing beta-galactosidase as an intracellular reporter, Peña et al. (2015) screened the GlaxoSmithKline diversity set of 1.8 million compounds. A total of 2310 compounds were identified with great potency against *T. cruzi* (pIC_50_ > 5) and a selectivity index > 10 [64]. The resulting lead compounds were further validated by Alonso-Padilla et al. (2015) using a novel, highly reproducible, high-content, high-throughput assay using myoblasts [65]. De Rycker et al. (2016) developed a new hit discovery screening cascade designed combining a primary imaging-based assay followed by newly developed and appropriately scaled secondary assays to predict the cidality and rate-of-kill of the compounds. This cascade was used to profile the SelleckChem set (421 FDA-approved drugs) and the NIH Clinical Collection set (727 compounds that have been used in clinical trials), leading to the identification of several known clinical compounds as candidates for a repurposing strategy for Chagas disease [66]. This cascade was further improved by the inclusion of three distinct in vitro assays: the slow replicating/cycling strain potency assay, the trypomastigote assay, and the extended duration washout assay [67]. Recently, Bernatchez et al. (2020) screened 7680 compounds from the Repurposing, Focused Rescue, and Accelerated Medchem library, and identified seven lead compounds with potent in vitro activity against *T. cruzi* and good therapeutic index [68]. 

## 3. Animal Models in Drug Discovery and Development against Trypanosomatids

Animal models are expected to mimic the pathophysiological features and immunological responses observed in the human host. A good experimental model for parasitic infections allows estimation of the specificity of drug action in relation to absorption, distribution, metabolism, excretion, and toxicity. Experimental models like rodents, dogs, and monkeys have been developed in order to identify and profile novel drugs against trypanosomatids, though mimicking the pathogenesis of disease and the impact of natural transmission is difficult to emulate under laboratory conditions [69]. The genotypic feature of laboratory models also augments hindrances due to restricted genotypic variations compared to infection with wild varieties. Hence, animal models developed and practiced for *T. brucei*, *T. cruzi*, or *Leishmania* infections do not accurately reproduce the consequences in human hosts, though several of these models exhibit an acceptable degree of proficiency for drug and vaccine development, particularly for the in vivo testing of trial compounds and libraries [70]. Important among them are BALB/c mice and Syrian golden hamster (primary tests), dogs (secondary tests), and monkeys (tertiary screens) as models for VL alongside athymic and SCID mice, which serve as a model for the treatment of VL in immunosuppressed conditions [69,71]. The genetic basis of the degree of susceptibility of mice to *Leishmania* has been linked to the Sc11 1a1 locus, based on which the outcome can be either self-healing or fatal [72]. The widely used (BALB/c and C57BL/6) mice breeds are mutated in the locus. In BALB/c mice, the immunopathology does not actually resemble human infection; instead, after around four weeks of infection, a strong Th1 response results in clearance of the parasite from the liver [72]. BALB/c is also highly susceptible to infection by *L. major,* with severe lesions and parasite-specific Th2 response with the enhanced expression of deactivating macrophage cytokines—particularly interleukin 4 (IL-4), interleukin 10 (IL-10), and transforming growth factor-β (TGF-β) [73]. On the contrary, the majority of inbred mouse strains like CBA and C57BL/6 are resistant to infection by *L. major,* and lesions spontaneously heal in 10–12 weeks [73]. The situation is bit different for the new-world *L. mexicana* and *L. amazonensis*, for which BALB/c, C57BL/6, and CBA/J mice are susceptible to infection [70]. On the contrary, for *L. braziliensis*, majority of mouse strains are resistant as the parasite does not induce protective Th2 response in the host [74]. However, for BALB/c, co-administration with salivary gland exudates of the vector promotes infection by altering the cytokine milieu [74]. Genetic susceptibility studies identified that the scl-1 locus controls the healing versus non-healing responses to *L. major* and the scl-2 is ascribed to the development of *L. mexicana*-induced cutaneous lesions. Around 30 loci have been identified as involved in the complex control of cutaneous leishmaniasis (CL) in mice [75]. BALB/c mice have been exploited as a model to profile metabolic changes during infection by *T. brucei* [72]. Mouse models including BALB/c, SCID, C57BL/6, and CH3 are the most widely used animal models in Chagas disease research [76]. However, the outcome was different in terms of Chagasic cardiomyopathy based on the strain of parasite and mouse line chosen for infection. Among alternative rodent models, guinea pigs have also been used as a model for experimental *T. cruzi* infection for acute and chronic Chagas disease [77,78,79]. For *T. brucei*, Wistar rats have been exploited as a preclinical model for HAT-associated cardiomyopathy [80]. The cotton rat (*Sigmodon hispidus*) represents one of the most susceptible animal hosts for *L. donovani*. The infection remains for 3–4 months, and after the appearance of initial clinical signs, the disease progresses rapidly, leading to death of the host [81]. Among various hamster species that are susceptible to *L. donovani*, the Syrian golden hamster (*Mesocricetus auratus*) represents a good model for VL with synchronous infection in the liver and spleen that culminates into a chronic non-cure infection with immune responses similar to human VL [81]. However, optimization of this model for drug screening is also effectively achieved through an ex-vivo splenic explant [45]. The only model that shows true potential for the evaluation of potential drugs targeting *L. braziliensis*, with low virulence for mice, is the golden hamster. Disease progression can be monitored over longer periods due to the chronic nature of the disease in the hamster [82]. For *L. infantum*, dogs are the natural reservoir. The natural infection of domestic dogs with *L. braziliensis*, *L. panamensis* and *L. mexicana* has been reported in Latin America. The infection of dogs with *L. infantum* is a pertinent laboratory model because it reproduces the natural infection with considerable similarity to human infections. The use of dogs as experimental models to study VL actually elucidated the role of immune cells, cytokines, and signaling events mediating immune response during *Leishmania* infection, offering crucial clues for developing immunotherapy. Canine models of *L. mexicana* infection have been established with Beagle dogs [83]. 

Non-human primates are exploited as the first experimental model for evaluating safety and efficacy of drugs and vaccines. For VL, *Macaca* sp. developed low and/or inconsistent infections. However, *Presbytis entellus* showed substantial susceptibility to hamster-derived amastigotes of *L. donovani* with all the clinical-immunopathological features as observed in kala-azar characterized by consistent and progressive acute fatal infection, leading to death between 110 to 150 days post-infection. The *L. major*–rhesus monkey model emulates self-limiting human cutaneous leishmaniasis that resolves within three months [73,84,85]. The model also shows promise in deciphering the intricacies of immune function and granuloma formation by *L. braziliensis*, rendering it as a useful model for drug and vaccine development [86]. Non-human primates have been explored as models for Chagas disease, but in most of the studied cases only a limited number of animals develop typical cardiomyopathy signifying *T. cruzi* infection [87]. Recent analysis of circulating leukocytes from naturally infected non-human primate cynomolgus macaque revealed a strong resemblance with immune-pathological biomarkers of Chagas disease in humans, projecting the prospect of this model in preclinical studies for new drugs for Chagas disease [87]. 

## 4. Cheminformatics in Drug Discovery

After the identification of several important and prospective drug targets like reductases of folate metabolic cascade, kinases, cAMP-phosphodiesterases, and enzymes for trypanothione synthesis and purine salvage, cheminformatics studies to identify structure–activity relationships for the design of optimized compounds have been prioritized. In recent times, combinatorial chemistry and HTS have enabled tests on large compound libraries, which encompass a significant chemical diversity, in short time scales [88,89]. Cheminformatics tools are broadly classified into structure- and ligand-based drug design (SBDD and LBDD) approaches. SBDD exploits the 3D coordinates of target structures for favorable ligand interactions. Potential ligands can be screened by molecular docking or structure-based virtual screening of potential ligands. High-affinity interactions between the binding site and ligand can be achieved by exploring binding site attributes like electronic distribution. The establishment of structure–activity relationships (SARs) can be achieved through experiments to further optimize ligand–receptor affinity [90]. Alternatively, ligand-based drug design studies can be performed without the receptor 3D structure. Instead, they require information on the structure, activity, and molecular properties of small molecules [91]. Chemometric models based on quantitative structure–activity and structure–property relationships (QSAR and QSPR, respectively) can be built in order to identify molecular descriptors complementing the target property [92].

Pteridine reductase (PTR1), an enzyme of the folate biosynthetic pathway, was one of the prominent candidates for drug targeting since no homologue of that protein is detectable in mammalian hosts. The crystal structure of LmjPTR1 was determined [93]. Implementing an SBDD strategy, Rasid et al. (2016) identified a number of dihydropyrimidine- and chalcone-based inhibitors for *Leishmania* PTR1 [94]. Using homology model for type 2 NADH dehydrogenase, Stevanovic et al. (2018) conducted a pharmacophore-based virtual screening to identify several hits [95]. A 6-methoxy-quinalidine derivative showed potential inhibition of the recombinant protein and inhibition of amastigotes with an EC_50_ of nanomolar range. Tryparedoxin peroxidase, a parasite-specific enzyme and a key component for parasitic survival under macrophage oxidative stress, has been considered as a key drug target. By performing deep molecular docking analysis with the crystal structure of PTR1 from *L. major*, a series of *N,N*-disubstituted 3-aminomethyl quinolones was identified which might serve as a worthy starting point for a suitable drug. SAR analysis of benzimidazole inhibitors against cysteine proteases cruzain and rhodesain from *T. brucei* and *T. cruzi*, followed by detailed cheminformatic analysis was conducted to find scaffold novelty and favorable physicochemical properties. Distinct endopeptidases like cathepsin-L-like CPB2.8 have emerged as exploitable drug targets in leishmaniasis. De Luca et al. (2018) identified a group of substituted benzimidazole derivatives that displayed strong (nanomolar) affinity for the protease from *L. mexicana* [96]. One of the compounds demonstrated a good bioavailability profile with ADMET analysis, implying it is a good future drug candidate. Carbonic anhydrases (CAs) have recently been identified from trypanosomatids. Cheminformatics analysis targeting this enzyme identified *N*-nitrosulfonamides as prospective inhibitors for CA from *Trypanosoma* and *Leishmania* over mammalian homologues. Being comparable with existing drugs in terms of EC_50_ and cytotoxicity, these compounds might serve as interesting leads for drug development. 

Using the ligand-based approach, aminophosphonates have been studied with QSAR modelling [97]. The authors took the gathered data for the whole compound series to build comparative molecular field analysis (CoMFA) models that suggested that several modifications can enhance the anti-leishmanial potential of α–aminophosphonates. Similar approaches identified 1,2,3-triazole and thiosemicarbazone hybrids and tetrahydro-β carboline derivatives as candidate anti-leishmanial drugs [98]. Novel quinazoline and arylimidamide derivatives have been identified using 3D QSAR-based analysis against *T. cruzi* [99]. The structure-guided discovery of a compound (compound 7) from the pyrazolopyrimidine series against a known protein kinase scaffold identified *Leishmania* CDK12 as a strong candidate for drug discovery. Structural studies combined to resistance mechanism analysis confirmed CDK12 as a specific target for the molecule [99]. With satisfactory specificity as well as pharmacokinetic and toxicological properties, the compound has been declared a preclinical candidate, suggesting cheminformatics can indeed boost systematic approaches to discover new drugs against trypanosomatids [99]. 

## 5. Quiescence, a Double-Edged Sword in the Quest of New Trypanocidal Drugs

Dormancy or persister cell formation is an evolutionarily conserved adaptive mechanism for stress tolerance for bacterial pathogens. Persister cell development is often associated with the development of a subset of a population that is metabolically quiescent and hence cannot be intervened by drug treatment [100]. Such an adaptation enables the parasite to survive under immunological stress and drug exposure, reverting to normal proliferative mode once the stresses disappear. Such conditions are well exemplified by the latent infection of *Mycobacterium tuberculosis* which can persist for the entire lifespan in a metabolically dormant state [101]. Similar metabolic diversions from proliferative to dormant state are observed in eukaryotic pathogens including fungal and parasitic protozoan infections [102]. The hypnozoite liver stages of *Plasmodium*, often associated with relapse of infection even years after successful therapeutic clearance, is one such persister-like stage for *Plasmodium vivax* [103]. For trypanosomatids, semi-quiescence to quiescence have been detected for intracellular forms of several species of *Leishmania* and in *T. cruzi* [102]. Persister formation is particularly relevant clinically for *Leishmania*, as relapsing conditions like post-kala-azar dermal leishmaniasis (PKDL) occurring several years after treatment for visceral leishmaniasis and leishmaniasis recidivans occurring after the treatment of cutaneous leishmaniasis emerge from possible metabolically distinct parasites that circumvent drug treatment due to dormancy without acquiring resistance by signature genetic alterations [104]. Despite its clinical significance, there has been a lack of concerted effort to study persister development in trypanosomatids due to technical constraints including the labelling of quiescent cells to distinguish them from the normally proliferating population. In 2015, a detailed identification and characterization of the semi-quiescent physiological state was reported in *L. mexicana* intracellular amastigotes in infected BALB/c non-healing lesions with a prolific increase in doubling time to ~12 days compared to ~4 days in ex-vivo macrophage infections [105]. The semi-quiescent metabolic state was also characterized by low rates of transcription and protein turnover that is distinct from stationary phase or metacyclic promastigotes, and is possibly a response to complex growth restriction in the intracellular microenvironment in granulomas. They identified two distinct macrophage populations, one with ~100 cells and the other with an average of ~400 intracellular amastigotes, suggesting the existence of two distinct metabolic amastigote varieties. *L. mexicana* amastigotes are intrinsically more resistant to nitric oxide and build up large communal phagolysosomes, while *L. major* infection is eventually controlled by an adaptive Th1 immune response requiring inducible NOS (iNOS) [105]. Mandell et al. (2015) identified a definite fraction of amastigotes with barely detectable replication in a C57BL/6J mouse model of cutaneous *L. major* infection. This population was observed to harbor in less-infected macrophages and constituted almost 39% of amastigotes under the persistent infection condition, while a second subset of amastigotes retained the ability to replicate with a doubling time of around 60 h [106]. *L. major* lacking the Golgi GDP-mannose transporter required for lipophosphoglycan synthesis encoded by LPG2 (lpg2-) persist in the absence of pathology, and in mouse infections this knocked-out line attained a persister-like feature immediately after infection [106]. *L. braziliensis* amastigotes (both axenic and intracellular) bear characteristic features of quiescence, with a radical reduction of (i) the kDNA mini-circle abundance, (ii) the intracellular ATP level, (iii) the ribosomal components, and (iv) total RNA and protein levels [107]. The untargeted metabolomic profile revealed the significant depletion of amino acids, polyamines, and trypanothione, with increases in ergosterol and cholesterol biosynthesis. Dormancy attains further relevance for trypanosomatid infection, as regimens including short-term therapy of even 60 days for *T. cruzi* infection is not related to resistance development, and the parasite possibly alleviates drug-mediated clearance by adopting quiescence. In fact, in *T. cruzi*, non-proliferating amastigotes develop both in vitro and in vivo models of infection. *T. cruzi* amastigotes regularly and spontaneously cease replication and become non-responsive to effective trypanocidal drugs like benznidazole and nifurtimox [108]. One or two such dormant parasites are detectable in each infected cell after treatment. Such dormant parasites reinitiate proliferation after drug withdrawal. Exploring the intricacies of the alteration of physiological status for intracellular amastigotes in infected tissues by proteomic or transcriptomic approaches is impaired by the paucity of enrichment protocols. Each of these studies adopted various strategies to characterize and label persister cells. One such strategy exploited ^2^H_2_O labelling for determining DNA, RNA, protein, and membrane lipids. The in vitro deuterium labelling of deoxyribose could be achieved for promastigotes by maintaining 5% ^2^H_2_O in medium, and for the in vivo labelling of amastigotes, 5% ^2^H_2_O in the body water was established by providing mice with a bolus of 100% ^2^H_2_O followed by inclusion of 9% ^2^H_2_O in the drinking water for up to several months [105]. Differential labelling for replicative and non-replicative amastigotes is achieved with CellTrace Violet or CellTracker Red. After a brief pulse, the stain is either diluted out during cell division (for replicative form) or remains at the initial pulse level (for non-replicating forms). This approach can be combined with a fluorescent (tdTomato) or luciferase expression system to track viable parasites [108]. The incorporation of thymidine analogues 5-ethynyl-2′-deoxyuridine and 5-bromo-2′-deoxyuridine has been implemented to differentiate replicative and non-replicative cells in *Leishmania* spp. and *T. cruzi* [108,109]. Each of these approaches has been effective in tracing persister cells. Active translation or ribosomal action utilizes 70% of the total ATP generated in a viable cell, and in quiescent cells translational activity is highly compromised, with a concomitant decrease in the number of active ribosomes (~5-fold reduction in dormant compared to normal metabolic state). Hence, the reduced transcription of rDNA loci serves as a marker for quiescence and rDNA loci are part of a rare genomic landscape in trypanosomatids, which is regulated by a definite transcription factor [110]. In this context, the expression of the GFP gene under the 18S ribosomal DNA locus has been implemented as a biosensor for quiescence in laboratory and clinical strains of *L. braziliensis* and *L. mexicana*, and reduction of GFP expression was compatible with BrdU uptake analysis in vitro. With this approach, a superior FACS quantitative approach for persisters could be devised for recording quiescence development in mice (BALB/c) or hamsters (LVG Golden Syrian Hamster) models [109]. The study provided a clearer idea about metabolic diversity in amastigotes with the coexistence of shallow and deep quiescent stages. Quiescence is crucial for subclinical infections with its potential role in drug tolerance, and quiescent cells serve as reservoirs for transmission and elicit a protective response against subsequent infections in trypanosomatids, which warrants additional exploration [106]. The development of novel assay methods combined with identification of strategies to combat dormancy or exploit it in developing immunization strategies might expedite the success of elimination programs against trypanosomatid parasites.

## 6. Cytology-Driven MoA Profiling

In the last few years, we have witnessed an increase in the number of scientific reports on new potential drug candidates to treat leishmaniases and trypanosomiases. However, the vast majority lack insights or detailed mechanism of action evidence supporting further drug development and clinical trials. In this scenario, cell-based assays offer the contextualized relevance and complexity of living cells to track drug discovery approaches, especially when considering unicellular parasites. Kinetoplastids are classified in this category due to the presence of a kinetoplast—a dense structure made by DNA (kDNA) within their unique mitochondria. Therefore, mitochondrial function monitoring can be applied in order to provide hints on the MoA of drug candidates in the drug discovery pipeline. Cellular bioenergetics analysis based on extracellular flux can phenotypically characterize mitochondrial function and define the energetic status of aerobic and glycolytic metabolism, defining a range from quiescent to energetic profiling [111,112]. This approach was used to monitor oxygen consumption (mitochondrial respiration) vs. medium acidification rate (glycolysis) in *L. infantum* to metabolically characterize SbR mutants and evaluate the oxidative role of gold(I) complexes as metallodrug candidates to treat leishmaniasis [44]. This approach was also considered using host cells experimentally infected with *T. cruzi* intracellular amastigotes, monitoring not only the parasite’s metabolism, but mimicking the natural conditions considering the context of endogenous conditions of infected cells [113]. These assays were performed on a Seahorse Extracellular Flux Analyzer, XF series (Agilent), and were initially used to monitor basal mitochondrial metabolism in *T. cruzi*, which is useful for drug screening purposes [114,115,116,117]. Microscopic imaging using cell-permeant mitochondrion-selective dyes such as MitoTracker or cell permeant acidotropic fluorophores like LysoTracker can be used to highlight ultrastructural alterations in essential organelles to make inferences about drug action and target elucidation by functional approaches [118]. These dyes can be used in high-content analysis approaches that have been shown as an alternative to monitor not only anti-parasitic drug action but also concomitant host toxicity analysis in the same assay for drug screening purposes [119]. Despite the above-mentioned fluorescent gene reporters, kDNA can be labelled to monitor cell replication for indirect drug activity measurement. The terminal deoxynucleotidyl transferase dUTP nick end labelling (TUNEL) technique allows the specific tagging of blunt DNA ends—a common feature in programmed cell death in mammalian cells. Conventional programmed cell death is not biochemically the same in trypanosomatids, and TUNEL signals are undetectable in trypanosome nuclei (genomic DNA). However, 25% of control (wild type, untreated) cells were reported to have TUNEL-positive kDNA. Treatments with eflornithine, nifurtimox, or melarsoprol did not change TUNEL signal, but pentamidine or suramin exposition reduced it, as an evidence of loss of kDNA following the latter treatments in a cell-cycle-dependent manner [120,121]. Trypanosomatids present closed mitosis (chromosomal condensation and segregation is maintained inside the nucleus during division), and the segregation of their single mitochondrial genome (kinetoplast) can be easily monitored by fluorescent microscopy during cell division in the presence of 4’,6’-diamidino-2-phenylindole (DAPI, a DNA-intercalating dye. This feature can be tracked under drug treatment to make inferences about mitosis or cytokinesis impairment. For example, non-treated *T. brucei* presented ~80% of cells with 1 nucleus and 1 kDNA pattern (1n1k), equivalent to G1 and S phase; ~15% were 1n2k (primarily G2 phase) and 5% were 2n2k (post mitosis). Suramin treatment switched profiling and 79% of the cells accumulated in >2n, indicating the blocking of cytokinesis in *T. brucei* [121]. A similar approach can be afforded using propidium iodide followed by flow cytometry analysis. Melarsoprol-treated *T. brucei* led to the accumulation of G2/M phase from 51% to 83%, indicating increasing replication but unsegregated nuclear genome, as an evidence of mitosis inhibition [121]. Genomic plasticity is a key factor in trypanosomatids, and plays an important role that must be taken into account when developing or testing new anti-trypanosomal drugs. In this context, DNA repair mechanisms are always being recruited, especially under stressful microenvironments like drug pressure. The enzyme uracil DNA glycosylase (UNG) participates in the DNA base excision repair (BER) pathway, and was found upregulated in *L. donovani* exposed to amphotericin B or sodium antimony gluconate. Curiously, drug-resistant clinical isolates of *L. donovani* from VL patients presented higher UNG expression [122]. suggesting that LdUNG plays a key role in BER, conferring moderate resistance to oxidants; this opens new avenues as a potential target for combination therapy against leishmaniasis. The adoption of drug discovery strategies against trypanosomatids must consider drug-resistance studies and the evolutionary role of DNA repair in this context. Antibodies can be used to track specific markers of DNA damage in eukaryotes such as the phosphorylation of threonine 130 at the C terminus of histone γH2A in *T. brucei*, which is associated with a delay in S and G2 phases of the cell cycle [123]. 

## 7. Genome-Wide Approaches in Target and Resistance (Resistomics)

Functional genomics approaches are useful for identifying or validating a given drug target. This relies on strategies or tools that can be combined together with studies on drug resistance mechanisms to find clues for drug discovery. For example, the in vitro selection of drug-resistant parasites, followed by whole-genome or transcriptomic sequencing could unveil targets or signatures associated with the drug used for resistance selection. This was the case of compound 7, DDD853651/GSK3186899, selected from a chemical series of pyrazolopyrimidine scaffolds, active against *T. brucei* and used to select resistant *L. donovani* mutants as a strategy to understand the MoA and to prospect potential pathways or drug targets [99]. Whole-genome sequencing of these drug-resistant parasites revealed a single homozygous non-synonymous mutation in CRK12 (cyclin-dependent kinase 12 or cdc-2-related kinase 12), leading to a Gly 572 to Asp in the predicted catalytic domain of the enzyme, impairing electrostatic interactions and causing resistance to the pyrazolopyrimidine [99]. In this case, the resistance mechanism identification was useful to pinpoint the drug target involved in drug action. Among trypanosomatids, *T. cruzi* and *Leishmania* species (the latter belonging to the *L. (Leishmania)* subgenus) lack one or more components of the RNA interference (RNAi) machinery. However, knockdown by RNAi manipulations can be performed in *T. brucei* and *L. (Viannia)* subgenus spp., a very useful functional genomic tool to validate and identify new drug targets [124]. Inspired by these biological features, Alsford et al. (2011) described a new technique called RIT-Seq (RNAi target sequencing), where *T. brucei* were transfected with a library of interfering RNAs able to silence >99% of the mRNA in the parasite. This was followed by culturing in the presence of drug pressure in which the recovered parasites had their enriched plasmids sequenced [125]. This functional cloning technique allowed a genome-scale knockdown profiling in which the decrease of a given gene product is selected as a phenotypical marker for surviving under a stressful condition. In this way, the mechanisms underlying selective drug action and resistance can be screened in a high-throughput genome-scale RNAi panel [126]. A phenotyping genome-scale RNAi screen revealed, for example, the involvement of aquaglyceroporin 2 (AQP2) in melarsoprol and pentamidine susceptibility in African trypanosomes [127,128]. Melarsoprol is an arsenic-based drug, and similar to antimony-based compounds against *Leishmania* parasites, is taken up through aquaglyceroporin 1, which was associated with antimony resistance by using a dominant negative functional cloning strategy using a cosmid library [129]. Cosmid libraries can also be applied to select gain-of-function genes associated with a given phenotype, where the screening is based on overexpressing libraries. This approach was used to confirm previous and pinpoint new drug resistance markers in *Leishmania* parasites—a technique called Cos-seq or cosmid-based functional screening coupled to next-generation sequencing [130,131]. The most recent brother of the X-Seq family is a technique called Mut-Seq, or chemical mutagenesis coupled to next-generation sequencing. In this case, “Darwinian hands play dice” leading to stochastic mutations that could be kept when important for parasite survival under stressful pressure. This was elegantly applied to study miltefosine and paromomycin resistance mechanisms in *Leishmania* parasites. After using Mut-Seq to identify new targets and validate the essential role of kinase CDPK1 on paromomycin resistance in *Leishmania* using CRISPR-Cas9, Bhattacharya et al. (2019) suggested that Mut-Seq screening is powerful tool to explore networks of drug resistance since CDPK1 was also involved in antimony resistance in the parasite [132]. Genome-wide approaches are very useful for capturing the main picture, and thus for choosing the most prominent biochemical pathway involved in drug action/resistance. This is also true when applying the revolutionary technique of genome editing: Clustered Regularly Interspaced Short Palindromic Repeats (CRISPR), CRISPR-associated gene 9 (Cas9)—CRISPR-Cas9. Beneke et al. (2017) developed a CRISPR-Cas9-based toolkit for the high-throughput genome editing of kinetoplastids that was further validated in single or multiple targets [133,134,135]. We are however currently revisiting concepts and moving from genome-wide approaches in parasite populations (or clones) to single-cell-based strategies to better understand the plasticity of *Leishmania* parasites that harbor mosaic aneuploidy—a feature that has impairments in the way the parasite will respond or not to a given drug. Using a single-cell genomic sequencing method, Negreira et al. (2020) identified 128 different karyotypes in 1560 *L. donovani* promastigotes [136]. They highlight the fact that some karyotypes presented pre-existing adaptations to antimony-based drugs, supporting a hypothesis raised even before this hint [137,138]. This reveals how complex it is to predict or open new avenues on MoA studies in trypanosomatids, and reinforces the evolutionary adaptions that guaranteed the establishment of trypanosomatids since the early Cretaceous [139]. Finally, and despite recent advances in genomic methods, there is still a relative paucity of functional annotations for a large number of gene products for trypanosomes, especially when compared to mammalian systems. In fact, this could explain why target-based methods lag behind phenotypic approaches in drug development for these parasites.

## 8. Metabolomics in Drug Screening

Like in a crime scene, studying the past is also a feasible alternative to tracking drug action and target identification. Metabolomics refers to the measurement of small metabolite molecules to investigate metabolic pathways, here in the context of drug discovery or target identification. Metabolite profiles are useful fingerprints offering clues on therapeutics targets in trypanosomatids, and can also be performed in the host to select signatures or markers associated with the dynamics of host–parasite interaction [140,141,142,143,144,145]. Metabolomics can also be applied to the rational development of defined minimal culture medium for in vitro drug screening purposes against trypanosomatids. In this regard, untargeted semi-quantitative or targeted quantitative metabolomics was used to decipher the major nutritional requirements of *T. brucei* and define all needs, removing unnecessary nutrients and improving drug sensitivity in activity studies [146]. Drug MoA can also be indirectly investigated through metabolomics, even without clear evidence on parasite alterations. Benznidazole is a 2-nitroimidazole prodrug that needs to be reduced in order to exert anti-trypanosomal activity against *T. cruzi*. Although benznidazole-treated parasites were minimally altered compared to untreated counterparts, metabolites concerning benznidazole linked to thiols such as trypanothione, glutathione, and cysteine indicates the thiol binding capacity of benznidazole on acting by disturbing redox homeostasis, leading to parasite death [147]. The cell redox system has also classically been related to antimony and resistance in *Leishmania* parasites. Combining untargeted metabolomics for initial screening coupled to ^13^C traceability assays, Rojo et al. (2015) confirmed and compiled multi-target metabolic alterations not only in redox, but also in detoxification, biosynthetic processes and amino acid metabolism in *L. infantum*. Antimony-resistant parasites presented incremented proline and glutamate, supporting previous reports on high levels of glycolytic markers in resistant *Leishmania* as revealed by proteomics [148,149]. In summary, metabolomics approaches helped to identify MoA or resistance of several anti-trypanosomal drugs such as eflornithine or halogenated pyrimidines against *T. brucei*; miltefosine and antimony against *Leishmania* parasites [150]. Drug targets can also be mined in trypanosomatids by metabolomics pathway analysis using in silico approaches, as a predictive way based on pathway annotation and searching for analogous or specific enzymes [151]. 

## 9. Theranostic Approaches

The term theranostic, derived from the fusion of the words therapeutic and diagnostic, is here used to define strategies designed for diagnostic purposes that also act as therapeutic agents. Dual-function molecules or smart probes can be adapted for both parasite detection/identification and anti-trypanosomatid activity. This combination of diagnosis and therapeutics is still a growing field and there are very few studies on trypanosomatids. A group headed by professors Eduardo Coelho and Luiz Ricardo Goulart in Brazil proposed the use of phage display—a high-throughput proteomic technology to generate and screen peptides and antibodies—for the serodiagnosis and prevention of leishmaniasis as a theranostic approach [152]. Using this approach, the team identified a β-tubulin from *L. infantum* that was highly antigenic and immunogenic, presenting good performance on diagnostic efficacy and eliciting Th1 response in vitro with high IFN-γ and low IL-10 levels [153]. Recently, Singh et al. (2019) reviewed the literature on nanomedicine-based approaches to circumvent leishmaniasis and concluded that much progress was made in the field reaching considerable milestones on VL nanomedicine, but translational research is needed for the coming decade for developing effective theranostic solutions [154]. Thus, many current alternatives such as liposomes, nanoemulsions, niosomes, nanodiscs, solid lipids nanoparticles, quantum dots, nanotubes, polymer conjugates, and inorganic compounds could be applied to clinical settings.

## 10. Case Study: Proteasomal Inhibitors against *Leishmania*

Proteasome targeted inhibitor developments by Khare et al. (2016) and Wyllie et al. (2018) are among the few major break-throughs in the quest of safe, easily deliverable, and selective drugs against trypanosomatids in recent times [155,156]. Both studies targeted the identification of a common target for intervention for *Leishmania* spp., *T. cruzi*, and *T. brucei* spp. Khare et al. began their screen with a library of 3 million compounds against the three pathogens, and identified an azabenzoxazole (GNF5343) that was effective against the three [155]. A number of substitutions leading to a less-toxic version GNF6702 further optimized the compound. In mouse model of VL and CL, with oral delivery of 10 mg kg^−1^ for eight days, GNF6702 caused significant amelioration of liver parasitic burden. Similarly, it displayed prolific attenuation of parasite load in mouse models of Chagas disease and HAT. For leishmaniasis, Chagas disease, and HAT, the activities are comparable to the approved drugs miltefosine, benznidazole, and diminazene aceturate, respectively. In fact, for HAT it performed better than the in-use diminazene aceturate in terms of diminishing parasitic infection in brain. The primary mechanism of parasite growth inhibition by the compound series was the selective inhibition of the proteasome chymotrypsin-like activity. For analyzing resistance against the drug, they raised mutants against an early version of the drug, which showed 40-fold lower susceptibility to the drug. The phenotype was attributed to a homozygous mutation in the proteasome β4 subunit (PSMB4I29M/I29M) and a heterozygous mutation (PSMB4wt/F24L). These mutations led to reduced susceptibility to inhibition by the drug. Interestingly, the chymotrypsic catalytic center is hosted by a β5 subunit and a β4 subunit in close contact with a β5 subunit forming a plausible binding pocket for the drug. The study suggested proteasomal subunits as a selective target for the development of a common chemical scaffold against trypanosomatids. In concordance, an independent screen by Wylie et al. identified and studied a second candidate GSK3494245/DDD01305143/compound 8 [156]. The precursor of the compound was developed by scaffold hopping and substitutions from a basic component identified by a phenotypic screen of around 16,000 molecules against *T. cruzi*, and demonstrated efficacy against intra-macrophage amastigotes of *L. donovani*. The compound showed good in vitro metabolic stability (CLint = 0.8 mL min^-1^ g^-1^) and selectivity over mammalian cells. They further addressed the compound in terms of duration of treatment by rate-of-kill assay that showed that induction of cell death is achievable within 72 h at nanomolar concentration range. Pharmacokinetic profiling for bioavailability and distribution revealed that it can be orally dosed to reach efficacious levels in a range of preclinical species, including mouse, rat, and dog. Moreover, virtually no significant safety or tolerability liabilities were detected by Ames test and in mouse lymphoma cells. For identifying the mechanism of action for the drug, the authors preliminarily adopted RIT-seq technology [125]. The study suggested that knock-down of nonessential genes of ubiquitination pathway rendered reduced sensitivity to the drug, pinpointing proteasome as the possible point of intervention for the drug. The generation of resistant mutants led to the identification of independent mutations in the β5 subunit (G197C and G197S). The mutants were cross-resistant to GNF6702. Both mutations affected proteasomal activity, as determined in vitro by UbiQ-018 label (a fluorescent label for proteasomal subunits), and the mutations resulted in insensitivity to GSK3494245 (compound 8). The proteasomal inhibitors caused cytological changes in *Leishmania* promastigotes with accumulation of vesicular structures and induced cell cycle arrest in G2/M phase. CryoEM of *L. tarentolae* proteasome in combination with compound 8 identified a number of residues from β4 and β5 subunits. Additionally, the selectivity of the drug for kinetoplastid proteasome over human proteasome could be attributed to a lack of hydrophobic interaction, as F24 in *L. tarentolae* corresponds to S23 in human and π-stacking interaction. Both works identified a suitable target for developing a common anti-trypanosomatid drug development and developed human-trial-ready molecules that precisely target chymotrypsin-like protease action of kinetoplastid proteasome without affecting the human orthologues.

## 11. Perspectives and Concluding Remarks

At present, drugs for treating trypanosomatid diseases are far from ideal due to host toxicity, elevated cost, limited access, and increasing rates of drug resistance. Therefore, new oral, safe, short-course drugs are urgently needed. Moreover, these new drugs have to be safe and effective enough to treat patients who are asymptomatic, as well as patients who develop secondary conditions such as post-kala-azar dermal leishmaniasis [14]. 

In the vast majority of cases, trypanocidal agents are out of the scope of interest of the pharmaceutical industry, mainly because it is unclear how to make a profit by selling them. This situation is also becoming more frequent in the case of the discovery and development of antibiotics [157]. For this reason, drug-discovery research of novel trypanocidal compounds has been traditionally fueled by non-profit and governmental organizations. However, in the last decade, some pharmaceutical companies have become more engaged and have joined forces with academia as well as governmental and non-profit organizations to tackle NTDs. This is the case of The Drugs for Neglected Diseases initiative (DND*i*), a nonprofit research and development organization founded by Médecins sans Frontières (MSF), among other public–private partners, which has campaigned for change since 2004 to raise awareness of the trypanosomatids crisis among key policy- and decision-makers [158]. DND*i* performs high-throughput untargeted screenings of novel-drugs libraries for trypanosomatids in addition to identifying new drug candidates using targeted compounds from repurposing libraries. Since its creation, DND*i* has already provided seven treatments: ASAQ and ASMQ (two fixed-dose antimalarials), nifurtimox-eflornithine combination therapy for late-stage sleeping sickness, sodium stibogluconate and paromomycin (SSG + PM) combination therapy for VL in Africa, a set of combination therapies for VL in Asia, and a pediatric dosage form of benznidazole for Chagas disease. While combination therapies will improve the efficacy of the treatment and reduce the emergence of drug-resistant strains, currently we do not have enough effective molecules to guarantee durable therapeutic strategies. Consequently, more efforts should be deployed to discover and exploit novel families of trypanocidal drugs (with different modes of action), which could be rapidly integrated in combinatory treatments, or kept as drugs of last resort when current combinations fail.

An important bottleneck in the discovery and development of new trypanocidal drugs is the lack of well-validated molecular targets, which has traditionally hindered the use of classic target-based approaches (usually applied to the discovery of antibiotics) in the drug-discovery cascade. While it is true that this has fostered the development and implementation of sophisticated phenotypic in vitro assays, these assays encompass major challenges specific to each parasite (e.g., drugs must be active in the phagolysosome milieu when treating patients infected with *Leishmania*, drugs for HAT have to cross the blood–brain barrier, etc.). Moreover, once a hit has been identified in a phenotypic screen, different approaches (e.g., genomics and proteomics) should be deployed to identify the specific target(s), mode-of-action of the compound, and to predict any potential mechanism of drug resistance deployed by the parasite. This information is crucial to guarantee a rational and successful optimization of the hit, and serves to develop novel target-based drug discovery cascades.

Another major challenge in drug discovery for trypanosomatids is the lack of well-defined standards/criteria (e.g., strain, culture media, incubation times, etc.) for the selection and validation of hit compounds, which sometimes leads to opposing results between different research teams. Among these criteria, one of the critical ones is the selection of the most relevant animal model that is able to mimic the pathophysiological features and immunological responses observed in human hosts (e.g., BALB/c mice vs. Syrian golden hamsters as models for *L. donovani* and *L. infantum*; acute vs. chronic models for Chagas disease, etc.). 

Moreover, in order to guarantee the success of drug discovery/repositioning in the fight against trypanosomatids, we have to generate high-quality data in many endemic countries (including field strains, drug-resistant strains, etc.), and to do so, we have to effectively increase the engagement of endemic countries in the R&D process [159]. 

New powerful and robust in vitro, in vivo, and in silico technologies have emerged in the last ten years. Moreover, we now have a more refined knowledge of the biology of these parasites, as well as the unprecedented ability to surgically manipulate trypanosomatids genome. The optimal use of these tools and knowledge will undoubtedly accelerate current drug discovery cascades, leading to the delivery of satisfactory treatment options for neglected patients with trypanosomatid infections.
